# A pilot study on factors linked to healthcare providers’ communication with older adults

**DOI:** 10.1590/1980-5764-DN-2025-0338

**Published:** 2026-03-23

**Authors:** Ouafa Izel, Halima El Houmaydy, El Mahjoub El Harsi, Abdelhafid Benksim, Abdelhamid Hachimi

**Affiliations:** 1 Cadi Ayyad University, Faculty of Sciences Semlalia, Department of Biology, Morpho-Science Research Laboratory, Marrakech, Morocco.; 2 Cadi Ayyad University, Faculty of Sciences Semlalia, Department of Biology, Laboratory of Pharmacology, Neurobiology, Anthropobiology and Environment, Marrakech, Morocco.; 3 Regional Health Directorate, Higher Institute of Nursing Professions and Health Techniques (ISPITS-M), Nursing Care Department, Marrakech, Morocco.; 4 Cadi Ayyad University, Faculty of Medicine and Pharmacy, Marrakech, Morocco.

**Keywords:** Communication, Aged, Caregivers, Aging, Comunicação, Idoso, Cuidadores, Envelhecimento

## Abstract

Effective communication between caregivers and older adults is a key element of optimal care, both therapeutically and psychologically. However, it can be hampered by a variety of factors relating to the older adults, the caregiver, and the working environment.

**Objective:**

The aim of this study was to assess the communication skills of caregivers with older adults and to identify the factors likely to influence them in the Marrakech-Safi region.

**Methods:**

A cross-sectional survey was conducted between April and October 2024 among 731 caregivers working in healthcare facilities in the Marrakech-Safi region. Caregivers’ communication was assessed using the Kalamazoo Essential Elements Communication Checklist–Adapted (KEECC-A). Multiple linear regression was used to determine potential factors influencing the KEECC-A score.

**Results:**

The mean overall KEECC-A score was 3.19±0.70. The lowest mean subscale scores were associated with reaching agreement (3.00±0.95), understanding the patient’s perspective (3.07±0.95), gathering information (3.13±0.94), and closing the interview (3.17±0.97). Multivariate analysis revealed that stress (B=-0.181±0.065; p=0.006) and illiteracy among older adults (B=0.147±0.057; p=0.010) were the main factors influencing caregivers’ communication skills.

**Conclusion:**

Although caregivers interact positively with illiterate older adults, stress has a negative impact on communication, highlighting the need for a holistic approach that considers both patient’s needs and caregivers’ work environment.

## RESUMO.

A comunicação eficaz entre os cuidadores e os pacientes idosos é um elemento fundamental para o atendimento ideal, tanto do ponto de vista terapêutico quanto do psicológico. No entanto, ela pode ser prejudicada por diversos fatores, relacionados ao paciente idoso, ao cuidador e ao ambiente de trabalho.

**Objetivo:**

O objetivo deste estudo foi avaliar as habilidades de comunicação dos cuidadores de idosos e identificar os fatores que podem influenciá-las na região de Marrakech-Safi.

**Métodos:**

Uma pesquisa transversal foi realizada entre abril e outubro de 2024 com 731 cuidadores que trabalham em unidades de saúde na região de Marrakech-Safi. A comunicação dos cuidadores foi avaliada usando a Lista de Verificação de Comunicação de Elementos Essenciais de Kalamazoo - Adaptada (KEECC-A), e a regressão linear múltipla foi empregada para determinar os possíveis fatores que influenciam a pontuação da KEECC-A.

**Resultados:**

A pontuação média geral da KEECC-A foi de 3,19±0,70. Os escores médios mais baixos da subescala foram associados à obtenção de concordância (3,00±0,95), à compreensão da perspectiva do paciente (3,07±0,95), à coleta de informações sobre ações (3,13±0,94) e ao encerramento da entrevista (3,17±0,97). A análise multivariada revelou que o estresse (B=-0,181±0,065; p=0,006) e o analfabetismo do paciente idoso (B=0,147±0,057; p=0,010) foram os principais fatores que influenciaram a qualidade da comunicação dos cuidadores com os idosos.

**Conclusão:**

Embora os cuidadores interajam positivamente com idosos analfabetos, o estresse prejudica negativamente a comunicação, destacando-se a necessidade de uma abordagem holística que considere tanto as necessidades dos pacientes quanto o ambiente de trabalho dos cuidadores.

## INTRODUCTION

 Effective communication between the caregiver and older adults is the cornerstone of a successful therapeutic relationship. Not only does it make it possible to respond to the specific needs of this category of person, but it also creates a climate of trust and reinforces therapeutic adherence through active and empathetic listening as well as verbal and nonverbal interactions^1^. However, ensuring quality communication remains a major challenge, as poor interaction can adversely affect patient therapeutic adherence and, consequently, the quality of care^2,3^. From this perspective, communication based on a patient-centered approach is essential. It presupposes the patient’s active involvement in the therapeutic process and requires caregivers to have specific communication skills, such as those described in the Kalamazoo Consensus^4^, which aim to establish a relationship of trust, allow the patient to express himself freely, collect information in a structured way using simple and understandable language, encourage the patient to ask questions, and involve him in decision-making^4-7^. Nevertheless, multiple factors can hamper the quality of communication, especially in geriatric care settings. These factors can be grouped into three main categories: those linked to the specific characteristics of older adults, those linked to healthcare professionals, and others linked to the environment and working conditions. Sensory impairments such as vision or hearing problems, cognitive problems, depression^8^, and socio-demographic characteristics such as age, gender, and level of education^9-11^, as well as language barriers^10-13^, are all factors likely to limit understanding and interaction between caregivers and older patients. In addition, certain socio-demographic variables among caregivers may also affect their ability to communicate effectively with older adults^14,15^. The care environment is also a major determinant. The stress^16-19^, the shortage of staff^20^, the multiplicity of tasks to be accomplished, the high number of patients to be managed^21,22^, and the lack of training in communication^23,24^ are all constraints likely to affect the quality of exchanges between caregivers and older adults. The aim of this study is to assess the communication skills of caregivers with older adults and to identify the factors likely to influence them. 

## METHODS

### Study design

 A cross-sectional survey was conducted between April and October 2024 among 731 physicians and nurses working in healthcare facilities in the Marrakech-Safi region, recruited through convenience sampling. 

#### Inclusion criteria

 All caregivers in direct contact with older adults. 

#### Exclusion criteria

 Pediatric and neonatal staff, medical students, and nursing students. 

### Data collection methods

 Data were collected using a self-administered questionnaire for caregivers. The questionnaire covered several dimensions, including the socio-demographic characteristics of caregivers, aspects related to older adults, and organizational elements likely to influence communication. 

 Aspects related to older adults included hearing, visuals, psychological and cognitive impairments, illiteracy, linguistic barriers, social isolation, gender, and socioeconomic status of the older adult. Organizational factors comprised the shortage of healthcare staff, perceived stress, training in caregiver communication, a wide range of tasks within the department, and the number of patients to manage. Caregivers’ communication was assessed using the Kalamazoo Essential Elements Communication Checklist–adapted (KEECC-A), a 7-item scale covering relationship building, initiating discussion, gathering information, understanding the patient’s perspective, information sharing, reaching agreement, and closing the conversation. The items were rated on a 5-point Likert scale ranging from 1 (poor) to 5 (excellent)^4-7^. A study conducted among physicians revealed that KEECC-A scores, obtained from professors, standardized patients, and physicians’ self-assessments, showed strong internal consistency and a stable single-factor structure^6^. Another study among healthcare professionals confirmed the validity of self-assessment using this instrument^25^. 

 Perceived stress among caregivers was measured using the Perceived Stress Scale version PSS-10. This tool is widely recognized for its validity, reliability, and adaptability to various contexts, including the healthcare field^26-28^. The PSS-10 scale consists of 10 items, rated on a Likert scale ranging from 0 ("never") to 4 ("very often"). It has a bifactorial structure, distinguishing between items formulated in a positive manner and those formulated in a negative manner. The total score can range from 0 to 40, with a score of 0–13 indicating low, 14–26 moderate, and 27–40 high perceived stress^26^. 

 In this study, Cronbach’s alpha coefficient was calculated for the KEECC-A and PSS-10 scales, yielding values of 0.88 and 0.74, respectively, indicating good internal consistency for both instruments. 

### Data analysis methods

 Data were examined using SPSS version 27.0. A one-sample Kolmogorov–Smirnov test was performed to assess the normality of continuous variables. Descriptive statistics including mean, standard deviation, and median were calculated to describe the KEECC-A score, and frequencies and percentages for categorical variables. 

 Associations between the KEECC-A score and explanatory variables were examined using the Student’s t-test, ANOVA, and the Welch test when variances were unequal. 

 Multiple linear regression was used to determine the potential factors influencing the KEECC-A score. Variables with a p≤0.10 in bivariate analyses were included in the multivariate model. Collinearity among the independent variables was assessed using the variance inflation factor (VIF). In the final model, predictors retained in the final model were considered statistically significant at p<0.05. 

 For certain variables, the total does not reach 731 due to missing values. These cases were excluded from the corresponding analyses. 

### Ethical considerations

 The ethical standards of the Declaration of Helsinki were respected during the study. The protocol was approved by the Ethics Committee of the University Hospital of the Marrakech-Safi region (N 94/2024), and informed consent was obtained from all participants before completing the questionnaire. The anonymity and confidentiality of the data collected were respected. 

## RESULTS

 A total of 731 caregivers were included in this study; the mean age of the participants was 32.95±8.39 years, with a predominance of females (72.6%); the majority of the participants came from urban areas (83.9%), were nurses (78.8%), and had been working between 5 and 15 years (50.3%) (Table 1). 

**Table 1:** Total score for caregivers’ communication skills according to their socio-demographic characteristics.

Variables		n (%)	Mean±SD	Statistical test	p-value
Age (years)					
	0–25	135 (18.7)	3.18±0.63	Welch’s ANOVA	0.130
	26–35	359 (49.7)	3.14±0.67		
	36–45	176 (24.4)	3.29±0.76		
	+46	52 (7.2)	3.29±0.89		
Gender					
	Male	200 (27.4)	3.28±0.75	Independent samples t-test	0.044
	Female	531 (72.6)	3.16±0.68		
Origin					
	Urban	614 (83.9)	3.18±0.70	Independent samples t-test	0.306
	Rural	117 (16.1)	3.25±0.71		
Marital status					
	With spouse	361 (49.8)	3.19±0.75	Independent samples t-test	0.997
	Without spouse	369 (50.1)	3.19±0.65		
Profession					
	Nurse	578 (78.8)	3.17±0.70	Independent samples t-test	0.054
	Physician	148 (20.4)	3.29±0.70		
Work experience					
	Less than or equal to 5	232 (31.7)	3.19±0.68	Welch’s ANOVA	0.017
	6–15	368 (50.3)	3.13±0.67		
	15 and over	130 (17.8)	3.36±0.81		

Abbreviation: SD, standard deviation.

 Descriptive analysis of the KEECC-A score revealed that the overall mean self-assessment of caregivers’ communication skills with older adults was 3.19±0.70. The lowest mean scores for the subscales were associated with obtaining agreement (3.00±0.95), understanding the patient’s perspective (3.07±0.95), gathering information (3.13±0.94), and closing the interview (3.17±0.97) (Table 2). 

**Table 2:** Total score and subscales of caregivers’ communication skills.

Communication skills	Mean±SD	Median
Builds a relationship	3.34±0.87	3.00
Opens the discussion	3.34±0.86	3.00
Gathers information	3.28±0.90	3.00
Understands the patient’s perspective	3.07±0.95	3.00
Shares information	3.13±0.94	3.00
Reaches agreement	3.00±0.95	3.00
Provides closure	3.17±0.97	3.00
Total score	3.19±0.70	3.14

Abbreviation: SD, standard deviation.

 Bivariate analysis showed statistically significant differences between caregivers’ communication skills and gender (p=0.044), years of work experience (p=0.017) (Table 1), communication training (p=0.008), stress (p= 0.000), the large number of patients to be managed (p=0.006), the multitude of tasks to be accomplished (p=0.021), the staff shortages (p=0.005) (Table 3), illiteracy among older adults (p=0.000), and language barriers (p=0.001) (Table 4). 

**Table 3:** Total score for caregivers’ communication skills as a function of factors linked to the work environment and conditions.

Variables			Mean±SD	Statistical test	p-value
A wide range of tasks within the department					
	Yes	501 (69.5)	3.16±0.68	Independent samples t-test	0.021
	None	218 (30.5)	3.29±0.74		
Large number of patients to manage					
	Yes	487 (67.5)	3.15±0.68	Independent samples t-test	0.006
	None	232 (32.5)	3.30±0.73		
Shortage of healthcare staff					
	Yes	354 (49.2)	3.12±0.73	Independent samples t-test	0.005
	None	365 (50.8)	3.27±0.67		
Stress related to work					
	Low stress	80 (11.5)	3.43±0.81	Welch’s ANOVA	0.000
	Moderate stress	597 (82)	3.18±0.69		
	High stress	47 (6.5)	2.89±0.58		
Training in caregiver communication					
	Yes	600 (83.2)	3.22±0.70	Independent samples t-test	0.008
	None	121 (16.8)	3.03±0.72		

Abbreviation: SD, standard deviation.

**Table 4:** Total score for caregivers’ communication skills as a function of characteristics related to the elderly.

Variables		n (%)	Mean±SD	p-value
Hearing impairment				
	Yes	612 (85)	3.20±0.70	0.990
	None	107 (15)	3.20±0.70	
Visual impairment				
	Yes	310 (43.1)	3.18±0.69	0.612
	None	409 (56.9)	3.21±0.71	
Cognitive impairment				
	Yes	484 (67.3)	3.19±0.69	0.857
	None	235 (32.7)	3.20±0.73	
Illiteracy				
	Yes	462 (64.2)	3.11±0.65	0.000
	No	257 (35.8)	3.36±0.76	
Psychological impairment				
	Yes	395 (54.8)	3.21±0.72	0.596
	None	324 (45.2)	3.18±0.68	
Linguistic barrier				
	Yes	374 (51.9)	3.11±0.65	0.001
	None	345 (48.1)	3.29±0.74	
Social isolation				
	Yes	205 (28.6)	3.12±0.64	0.054
	None	514 (71.4)	3.23±0.72	
Elderly female				
	Yes	87 (12.1)	3.13±0.62	0.350
	None	632 (87.9)	3.21±0.71	
Elderly male				
	Yes	83 (11.5)	3.19±0.72	0.928
	None	636 (88.5)	3.20±0.70	
Low socioeconomic status				
	Yes	248 (34.5)	3.16±0.71	0.310
	None	471 (65.5)	3.22±0.70	

Abbreviation: SD, standard deviation.

Note: Statistical test: independent samples t-test.

Table 5 presents the multivariate analysis of factors associated with caregivers’ communication skills. The multiple linear regression model identified illiteracy among older adults and stress as significant predictors of caregivers’ communication. Increased stress levels among caregivers were associated with lower communication scores (B=-0.181±0.065; p=0.006). The standardized coefficient (β=-0.108) indicates that the effect, while negative and statistically significant, is relatively small. Contrary to our expectations, illiteracy among older adults was positively associated with caregivers’ communication skills (B=0.147±0.057; p=0.010), with a (β=0.100) indicating a modest effect. This suggests that, among all variables in the model, stress and illiteracy, which have the highest standardized β coefficients, exert the greatest influence on communication, although the absolute effect remains modest. No collinearity detected (VIF<2 for all predictors). 

**Table 5:** Variables associated with the total score of caregivers’ communication skills according to the multiple linear regression model.

Variables	B±SE	β	p-value
Genre	0.059±0.060	0.037	0.331
Work experience	0.040±0.038	0.039	0.290
Illiteracy	0.147±0.057	0.100	0.010
Linguistic barrier	0.092±0.054	0.065	0.087
A wide range of tasks within the department	0.077±0.059	0.050	0.193
Large number of patients to manage	0.055±0.058	0.036	0.347
Shortage of healthcare staff	0.103±0.053	0.073	0.054
Stress related to work	-0.181±0.065	-0.108	0.006
Training in caregiver communication	-0.137±0.071	0.072	0.055

Abbreviations: B, regression coefficient; SE, standard error; β, standardized coefficients.

Note: Statistical test: multiple linear regression.

## DISCUSSION

 The aim of the present study was to assess caregivers’ communication with older adults and to identify the factors influencing it in the Marrakech-Safi region. The results revealed a low self-assessment of caregivers’ communication skills, with an average score lower than those reported in other studies^25,29^. This finding suggests a limited perception of their own communication abilities, reflecting a deficit that warrants particular attention. These results are consistent with a previous study conducted in the same region, which highlighted the dissatisfaction among older adults regarding caregivers’ communication^8^. 

 A detailed analysis of the communication scale revealed notable deficiencies, particularly in obtaining agreement, understanding the patient’s perspective, gathering information, and closing the interview (Table 2). These findings are supported by studies conducted in China, which showed that caregivers’ low communication skills, especially in understanding the patient’s viewpoint and properly concluding the consultation, contribute to patients’ dissatisfaction^30^. 

 The communication deficits identified among caregivers may be explained by a combination of factors related to their individual characteristics, the specific needs of older patients, and the environmental and organizational constraints of the care setting. 

 Bivariate analysis revealed a significant association of socio-demographic characteristics (Table 1), notably gender and work experience, with caregivers’ communication skills. Although studies have shown that women have higher communication skills, based on empathy, listening, and emotional involvement, whereas men prefer more direct communication, focused on technical skills^15^. Our study revealed contradictory results; this discrepancy could be explained by a tendency for men to overestimate their own skills, while women, often more demanding of themselves, are often more critical and may underestimate their abilities, even when their actual skills are similar. These subjective perceptions could represent a social desirability bias. 

 The study also revealed a positive association between caregivers’ professional experience and their level of communication, a finding that corroborates the results of research carried out with older patients in long-term care, highlighting that more experienced caregivers are more aware of the cognitive and sensorimotor changes in older adults, induced by aging, which frequently hinder communication^14^. With the experience acquired over time, caregivers are able to adopt appropriate strategies that enable more efficient and effective exchanges, contributing to a better understanding and satisfaction among older adults. 

 The study also showed an association between caregiver communication and factors related to the working environment and conditions (Table 3). These factors include staff shortages, high workloads marked by large numbers of patients to care for and multiple tasks, as well as communication training and stress levels. One study has underlined the negative impact of the shortage of nursing staff on the quality of care and patient safety^20^, which supports the results of the present study. The problem of the shortage of nursing staff is even more striking in Morocco^29^, further amplifying the work overload. Caregivers are forced to manage a multitude of tasks and a large number of older adults simultaneously. Several studies have reported that excessive workload is strongly associated with reduced quality of care, particularly with regard to caregiver–patient interactions^21,22^, which corroborates our results. Indeed, having to manage several tasks at the same time leads to a loss of concentration on the part of caregivers and increases the risk of errors and oversights. Lack of time often prevents caregivers from adopting a holistic approach to care^31^. Further, training is the lever for professional development, particularly in the healthcare field, as it enhances caregivers’ skills. The present study demonstrated a significant association between caregivers having acquired communication training and the required communication skills. This finding is in line with other studies that focus on the important role of communication training in contexts where patient’s needs are complex, showing that training improves caregiver efficiency, patient satisfaction, and, consequently, overall quality of care^23,24^. 

 Bivariate analysis showed an association between these factors and the KEECC-A score; however, this association disappeared in the multiple linear regression, which revealed that stress and illiteracy are the potential predictive factors influencing the quality of communication between caregivers and older adults. Although the magnitude of their effect appears modest, these two variables present the highest standardized coefficients among all the factors included in the model, underlining their relative importance. Nevertheless, the weakness of these coefficients suggests that communication is a multifactorial process, also influenced by other variables that were not included in the model (Table 5). 

 Illiteracy is often seen as a factor inhibiting communication^7,11^. Contrary to our expectations, the study revealed a slight improvement in caregivers’ communication with illiterate patients, as a result of caregivers’ awareness of the specific needs of illiterate older adults. This finding is of particular importance in the Moroccan context, according to the Moroccan National Survey on Population and Family Health, 71.6% of people aged 60 and over are illiterate^32^. This prevalence may encourage healthcare professionals to make greater efforts and adapt their communication accordingly, using strategies such as simplifying language, reformulating messages, clarifying medical terminology, translating technical terms or specific concepts into the local dialect, employing nonverbal communication (including touch), and using visual aids to enhance patients’ understanding of the information provided^1,12^. 

 Moreover, stress represents a major challenge for healthcare professionals^33^. The present study demonstrated the negative impact of occupational stress on the quality of communication between caregivers and older adults. This conclusion supports previous studies, which emphasize that stress can lead to reduced alertness, decreased job satisfaction, and communication difficulties. These effects have significant repercussions not only on the quality of care provided to patients but also on the well-being of the caregivers themselves^16-19^. Indeed, stress depletes the physical and emotional resources of caregivers, limiting their ability to listen, understand, and interact with older adults^34^, who often present with sensory impairments, speech disorders, and cognitive and psychological deficits, making communication particularly complex^8^. In this context, caregivers tend to favor brief and low-empathy interactions focused on performing technical tasks, to the detriment of the relational dimension of care. This dynamic can lead to a form of relational disengagement, marked by avoiding direct interaction with older adults, delegating communication to companions or family members, and giving only limited consideration to patients’ autonomy, which raises important ethical concerns. This lack of direct communication, combined with a performance-centered approach to care, can result in misunderstandings, frustration, and communicative violence, ultimately compromising the caregiver–-elderly patient relationship and potentially impacting the quality and safety of care^35-37^. 

 Nevertheless, despite the efforts and strategies deployed by healthcare professionals to adapt their communication, implementation remains complex due to the specificity, complexity, and precision of medical terminology. This can lead to ambiguities and confusion among patients and generate additional stress for the caregiver, who must make extra efforts to ensure better understanding^1,12^. 

 The implications of this study are multiple and hold significant importance for geriatric practice, health sciences research, and health policy development. By highlighting the considerable impact of stress on communication with older adults, these findings provide strong arguments in favor of policies aimed at reducing workload and strengthening caregiver staffing, thereby improving both caregiver well-being and the quality of care provided to older adults. This underscores the importance of enhancing initial and continuing training for caregivers in geriatric communication and personal development, particularly stress management. Furthermore, the establishment of regional structures specialized in comprehensive care for older adults is a priority, especially in the context of a growing aging population. These structures should adopt a multidisciplinary approach involving physicians, nurses, psychologists, occupational therapists, physiotherapists, and social workers to ensure person-centered care. It is also essential to adapt the hospital environment by implementing clear and accessible visual signage, as well as therapeutic education units that consider limitations related to illiteracy. Future research using objective measures should clarify whether the observed protective effect of illiteracy reflects a perceptual bias or a genuine adaptive mechanism, using objective measures. 

 This study has certain limitations. On the one hand, it relies exclusively on self-assessment of healthcare professionals’ communication skills, which may introduce a social desirability bias. On the other hand, the wide range of age groups among the patients being cared for leads healthcare professionals to adopt generalized and uniform communication strategies, due to the lack of healthcare structures specifically dedicated to older adults at the regional level and the shortage of professionals with specialized training in geriatrics. And finally, convenience sampling limits the ability to generate results for the entire population. 

 In conclusion, this study highlights the critical importance of effective communication between caregivers and older adults. The assessment of caregivers’ communication skills revealed significant gaps, particularly in reaching agreement, understanding the patient’s perspective, gathering information, and closing the interaction. Caregiver stress negatively impacts communication, while illiteracy among older adults appears to have a protective effect. Despite caregivers’ efforts to adapt their communication, these strategies do not fully compensate for emotional overload and work-related stress. Strengthening targeted training, multidisciplinary teams, and adapting the hospital environment are essential measures to ensure high-quality care tailored to the needs and specificities of older adults. 

## Data Availability

The datasets generated and/or analyzed during the current study are available from the corresponding author upon reasonable request.
